# Ecological Momentary Assessment Within a Digital Health Intervention for Reminiscence in Persons With Dementia and Caregivers: User Engagement Study

**DOI:** 10.2196/17120

**Published:** 2020-07-06

**Authors:** Courtney Potts, Raymond Bond, Assumpta Ryan, Maurice Mulvenna, Claire McCauley, Elizabeth Laird, Deborah Goode

**Affiliations:** 1 Ulster University School of Computing Jordanstown United Kingdom; 2 Ulster University Institute of Nursing and Health Research Derry United Kingdom

**Keywords:** ecological momentary assessment, EMA, app, behaviour analytics, event logging, dementia, carers, reminiscence, reminiscing, mHealth

## Abstract

**Background:**

User-interaction event logs provide rich and large data sets that can provide valuable insights into how people engage with technology. Approaches such as ecological momentary assessment (EMA) can be used to gather accurate real-time data in an individual’s natural environment by asking questions at any given instant.

**Objective:**

The purpose of this study was to evaluate user engagement and responses to EMA questions using InspireD, an app used for reminiscence by persons with dementia and their caregivers. Research findings can be used to inform EMA use within digital health interventions.

**Methods:**

A feasibility trial was conducted in which participants (n=56) used the InspireD app over a 12-week period. Participants were a mean age of 73 (SD 13) and were either persons with dementia (n=28) or their caregivers (n=28). Questions, which they could either answer or choose to dismiss, were presented to participants at various instants after reminiscence with personal or generic photos, videos, and music. Presentation and dismissal rates for questions were compared by hour of the day and by trial week to investigate user engagement.

**Results:**

Overall engagement was high, with 69.1% of questions answered when presented. Questions that were presented in the evening had the lowest dismissal rate; the dismissal rate for questions presented at 9 PM was significantly lower than the dismissal rate for questions presented at 11 AM (9 PM: 10%; 11 AM: 50%; χ^2^_1_=21.4, *P*<.001). Questions asked following reminiscence with personal media, especially those asked after personal photos, were less likely to be answered compared to those asked after other media. In contrast, questions asked after the user had listened to generic media, in particular those asked after generic music, were much more likely to be answered.

**Conclusions:**

The main limitation of our study was the lack of generalizability of results to a larger population given the quasi-experimental design and older demographic where half of participants were persons with dementia; however, this study shows that older people are willing to participate and engage in EMA. Based on this study, we propose a series of recommendations for app design to increase user engagement with EMA. These include presenting questions no more than once per day, after 8 PM in the evening, and only if the user is not trying to complete a task within the app.

## Introduction

### Background

Digital health and well-being products such as health apps are becoming increasingly popular given that mobile technology is ubiquitous in daily life. In addition to the user data that are recorded during use of these platforms, all user interactions and events can be logged to represent usage. Such user interaction and event logs provide rich and large data sets that can reveal valuable insights into how people engage with technology. This case study explored user engagement with ecological momentary assessment (EMA) in an app designed for persons with dementia and their caregivers.

Dementia includes a group of symptoms associated with ongoing cognitive decline and is highly prevalent with approximately 50 million cases worldwide [1]. Within the United Kingdom, 1 in 6 people over the age of 80 have dementia, and the incidence of dementia is projected to double by the year 2050 [2]. Dementia also has a large economic impact; it is estimated to cost £26 billion (approximately US $33 billion) a year in the UK alone [3]. Currently, there is no treatment that can prevent, cure, or slow the progression of dementia. Pharmacologic treatments such as antipsychotic medications have been used to treat the symptoms of dementia but with limited success and unwanted side effects [4]; therefore, nonpharmacologic interventions such as reminiscence are increasingly considered in dementia care. Reminiscence has been defined as recall of events in a person’s life either individually or with others [5]. The process of reminiscence can involve the use of prompts such as photographs, music, and videos to trigger memories that have a special meaning for a person. Engaging in reminiscence increases sociability, confirms personal identity, and encourages feelings of self-worth [6,7]. A recent review [8] found evidence that reminiscence for those living with dementia helped to enhance quality of life in care homes and benefited those who felt depressed in an individual setting.

Many different techniques (such as EMA or experience sampling methodology) can be used to gather accurate data on daily living. These methodologies are used to capture real-time data in an individual’s natural environment through repeated sampling [9]. This can include psychometric scales, open-ended questions, or anything else that can be used to assess an individual’s condition at any given place and time. These approaches provide a high degree of ecological validity as they study people as they go about their day-to-day lives [9]. Since EMA requires that participants respond to questions at a given moment, it avoids recall bias which makes it a useful tool for those with memory impairment (such as persons with dementia). Traditionally, EMA used paper diary techniques, but now, devices such smartphones or tablets can be used to record digital data. Recently, a study [10] used EMA to identify major areas of concern for caregivers of persons with Alzheimer disease; the study’s overall goal was to provide support and information for caregivers in their home. Another study used experience sampling methodology to examine the day-to-day effects of caregiving on dementia caregivers which could be used to tailor interventions to their individual needs [11]; however, there has been a paucity of research to date examining the use of EMA to sample data from both persons with dementia and their caregivers.

### Previous InspireD Study

In our previous work, we developed an app for reminiscence which incorporated EMA for the Individual Specific Reminiscence in Dementia (InspireD) feasibility study. The study used a quasi-experimental design and investigated the use of an iPad app to allow people living with mild to moderate dementia and their family caregivers to reminisce (Multimedia Appendix 1). In phase one, a group of volunteers which included caregivers and persons with dementia cocreated, refined, and developed the app for InspireD. The app allowed users to electronically collect and store personal and generic media in the form of music, photographs, and videos. Phase two involved implementation which included training and use of the app with persons with dementia and their caregivers. Participants used the app at home for a period of 12 weeks. The primary outcome measure was the impact of reminiscence on mutuality [12] defined as the positive quality of the relationship between caregiver and care receiver. The secondary outcome measures included well-being measured using the 5-item World Health Organization Well-Being Index [13] and quality of the relationship between the person living with dementia and their caregiver using Quality of the Carer-Patient Relationship [14]. The third and final phase included individual interviews with participants to explore individual views on the intervention.

Reminiscing made up the largest proportion of total app interactions for persons with dementia (71%) and their caregivers (47%) [15]. Across both groups, there were more interactions with photographs than interactions with music and video [15]. The app was primarily used for reminiscence using personal multimedia content as opposed to generic photos and videos. [15]. The most popular times to use the app were around 11 AM, 3 PM, and 8 PM corresponding to postbreakfast, postlunch, and postevening mealtimes [15]. On average, a person living with dementia used the app about once per week over the 12-week trial period [15].

Participants living with dementia attained statistically significant increases in mutuality, quality of caregiver and patient relationship, and subjective well-being from the beginning to end of the study [16]. Additionally, unsupervised machine learning was used to identify behavioral clusters that characterized different user engagement with the InspireD app which were cross compared with qualitative data following interviews after the trial period [17].

### Objectives

In this study, we sought to address four research questions: (1) What is the temporal engagement with EMA questions over hours of the day and across the trial period? (2) How differently do persons with dementia engage in responding to questions compared to how caregivers engage? (3) How do persons with dementia and their caregivers engage in responding to questions after reminiscing with video, audio, and photos? (4) How do persons with dementia and their caregivers engage in responding to questions after reminiscing with personal media compared to how they respond after reminiscing with generic media? The aim of this study was to evaluate engagement with EMA questions while using an app with the overall goal to inform EMA use within digital health interventions.

## Methods

### Study Design

A user engagement study was conducted in which participants used a digital health intervention, an app for reminiscence, at home for a trial period of 12 weeks. EMA questions on mutuality were presented to users at various points during the trial period while they were reminiscing using the app.

### Participants

The study received ethical approval in March 2016 (16/NI/0035) in line with regional and national Health Service Trust research governance. Ethical considerations were ensuring voluntary participation by supporting separate informed consent for the persons with dementia and their caregivers, secure handling and storage of data in line with University policy, and reminding participants of their right to withdraw from the study at any time. In this study, for each person with dementia who participated, a family caregiver also participated. Each dyad (person with dementia and their caregiver) was given an iPad and asked to use the app at home for a 12-week period. Participants were encouraged verbally and in writing to use the app at least 3 times per week during the trial period. Information technology training was provided on 3 occasions; 2 sessions were provided before the trial period began, and 1 session was provided in week 6 of the trial period. The purpose of the training was to provide guidance on how to use the app, how to upload media, and to provide general app support. The app recorded user event logs locally on each iPad using an SQLite database (a public domain structured query language database). User event log data were later collected in person from the iPads using a portable storage device. Specific activities and activity types were logged: entry (logging in), administrative activities (adding or deleting photos, videos, or music), reminiscing (viewing photos, videos, or listening to music), queries at a given instant (EMA questions), and exiting (logging out). The app allowed individuals to upload their own photos, videos, and music or to access media online. For the purposes of this study, generic media was defined as photos, videos, and music that were accessed online through the app, for example, a photograph of street where the individual lived as a child. Personal media was defined as media that was uploaded, such as an old family photograph.

### EMA

**Figure 1:**
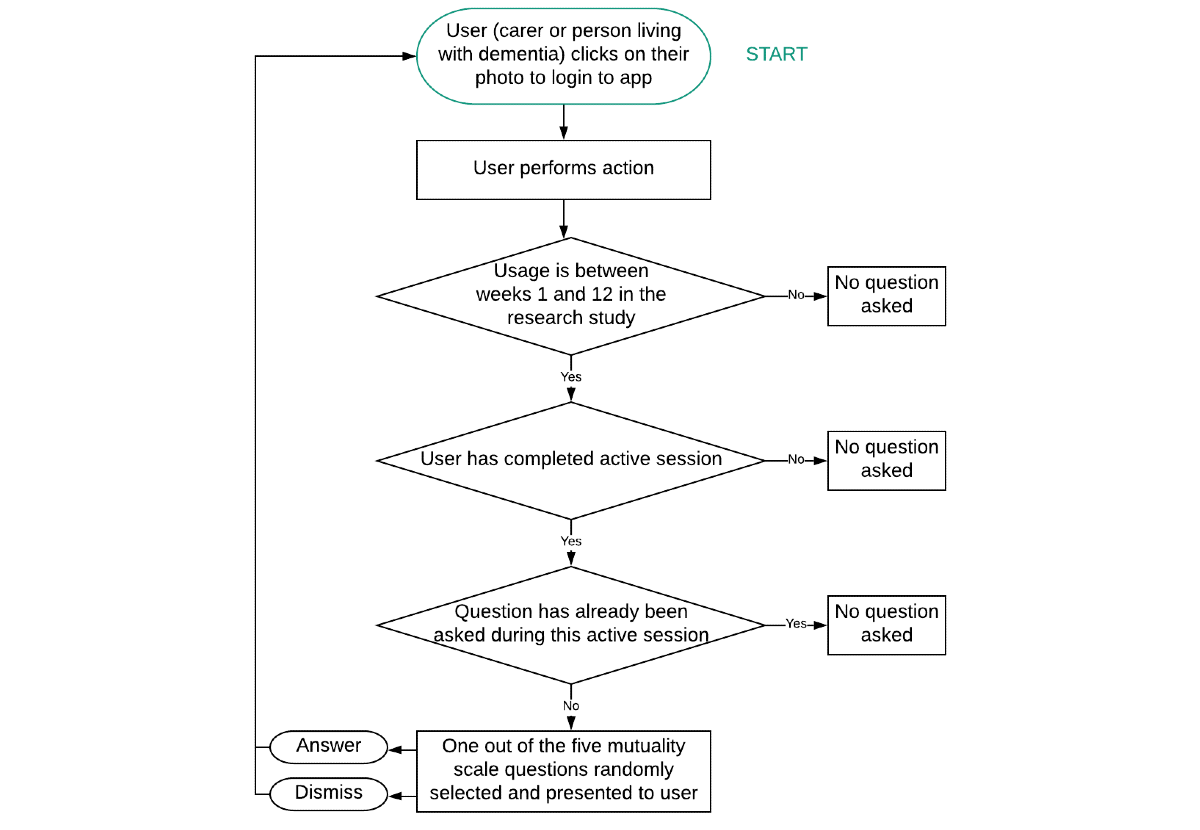
Logic flowchart for ecological momentary assessment questions presented to people living with dementia and their caregivers (carers). If these conditions are met, then a question is presented to the user who answers or dismisses the question. Subsequently, the loop begins again.

The EMA questions were a subset of items derived from the Mutuality Scale [12]. Over the 12-week trial period, the EMA questions were presented at random while individuals were using the app; therefore, questions were only displayed when the app was already in use. Hence, no push notifications were sent when the app was not in use to encourage completion of EMA questions. While the user was carrying out an action within the app (Multimedia Appendix 2), a series of checks were carried out before an EMA question was displayed (Figure 1). The user could either choose to answer the question using 5-point Likert scale (a great deal, quite a bit, some, a little, not at all) or choose to dismiss the question. This study did not analyze the responses that were provided, only whether the user chose to answer or dismiss the question.

### Data Analysis

R studio (version 3.6.0) and the R programming language were used for data wrangling and statistical programming. For the first part of the analysis, the user log data were filtered to only contain data related to queries at a given instant in order to look at overall engagement with questions. Over the course of the 12-week trial period, a total of 832 questions were presented. Of these questions, 77 were asked during training days and so were excluded from this analysis, leaving 755 questions asked during the trial period.

Data were aggregated by time of day (hourly), and by week (weeks 1 to 12) to obtain question presentation counts and dismissal rates for each user type (persons with dementia or caregivers). Data were aggregated over the 12-week trial period to obtain total question presentation counts and dismissal rates for each user type (persons with dementia, or caregivers), media format (photo, video, music), media source (personal or generic), and the last logged activity (log-in, add or delete photo, exit app). Some media could not be categorized into generic or personal media and were, therefore, only included in the aggregate media category (all). For last logged activity, 124 out of 628 (16%) EMA questions were filtered out of the data since the previous recorded event had been an EMA question and not an action that could trigger a question (Multimedia Appendix 2).

Spearman rank correlation coefficients were used for association analysis between the number of questions presented and the number of interactions with the app, number of app interactions and the number of questions presented across trial weeks, dismissal rate and time of day, dismissal rate and trial week, number of questions presented and the number of questions dismissed per hour, where *P*<.05 was considered statistically significant. Chi-square tests were used to compare the proportion of questions answered versus dismissed across hours of the day, across trial weeks and for other logged activities—log-in, add or delete photo, exit app. Pairwise chi-square tests (n=36) adjusted for multiple testing using Bonferroni correction were used to compare dismissal rates for each question following reminiscence with all photos, videos and music, and then segregated into personal and generic photos, videos and music (α=.05/36; therefore *P*<.001 was considered statistically significant).

## Results

### Participants

A total of 30 dyads (person with dementia and their caregiver) were recruited to the study; however, usage data was found to be corrupted in the iPad software used by 2 participants with dementia and their caregivers. Therefore, only 28 sets of tracking data (n=56 participants) were analyzed in this study. The characteristics of the participants are shown in Table 1.

**Table 1:** Characteristics of participants in the InspireD study.

Characteristics		All participants (N=56)	Persons with dementia (n=28)	Family caregivers (n=28)
**Age (years)**				
	mean (SD)	73 (13)	79 (12.1)	67 (13)
	range	31-94	61-94	31-91
**Gender, n (%)**				
	Male	24 (43)	18 (64)	6 (21)
	Female	32 (57)	10 (36)	22 (79)
**Previous information technology experience, n (%)**				
	Little or none	33 (59)	23 (82)	10 (36)
	Some	19 (34)	4 (14)	15 (53)
	A lot	4 (7)	1 (4)	3 (11)

### General Engagement

Five different EMA questions from the Mutuality Scale [12] were presented to persons with dementia and their caregivers (Table 2). There was a significant correlation between the number of questions presented and the number of interactions with the app (ρ=0.86, *P*<.001), with roughly 1 question asked for every 10 app interactions. The overall dismissal rate for questions asked during the trial period excluding training days was 30.9%. Hence, 522 out of 755 (69.1%) questions were answered. Persons with dementia used the app more in the trial period than caregivers did [15], but despite this had a lower dismissal rate for questions (121/451, 26.8%) compared to that of their caregivers (112/304, 36.8%).

A breakdown of presentation and dismissal for each question is shown in Table 2 along with chi-square test results comparing the proportion of questions answered versus dismissed. The dismissal rates were significantly different from the answer rates for each question (Table 2).

**Table 2:** Ecological momentary assessment questions presented and dismissed.

Questions		Presented, n (%)	Dismissed, n (%)	Chi-square (*df*)	*P* value
1	How attached are you to {partners name}?	197 (26.1)	48 (20.6)	101.5 (1)	<.001
2	How much do the two of you laugh together?	131 (17.4)	39 (16.7)	30.9 (1)	<.001
3	How much do you confide in {partners name}?	116 (15.4)	38 (16.7)	17.7 (1)	<.001
4	How much do you enjoy sharing past experiences with {partners name}?	165 (21.9)	54 (23.2)	32.7 (1)	<.001
5	How much do you like to sit and talk with {partners name}?	146 (19.3)	53 (22.8)	16.6 (1)	<.001

### Engagement by Time of Day and by Week

**Figure 2:**
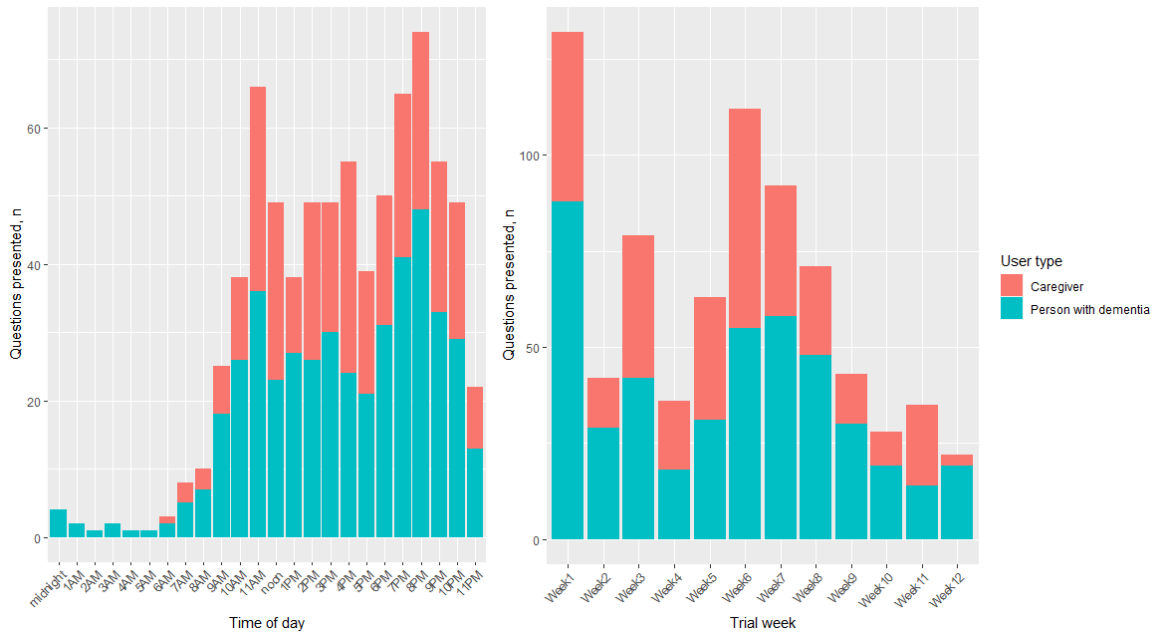
Total number of questions presented across hours of day (left) and trial weeks (right) for each user type.

The number of questions presented per hour and per week are shown in Figure 2. The fewest number of questions were presented between midnight and 8 AM (Figure 2). Most questions were presented late morning (between 10 AM and noon), late afternoon (2 PM to 4 PM), and after dinner (7 PM to 9 PM). A high number of questions were presented in weeks 1 and 6, which is probably due to increased use of the app post–information technology training and was consistent with increased app usage in weeks 1 and 6. There was a significant association between the number of app interactions and the number of questions presented across trial weeks (ρ=0.84, *P*=.001).

**Figure 3:**
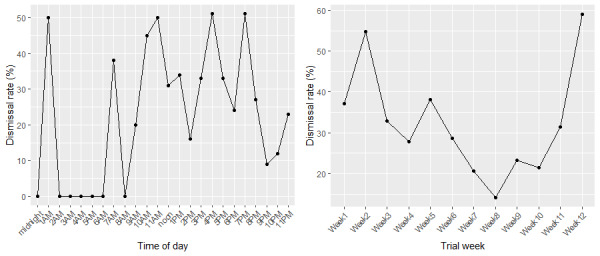
Dismissal rate for ecological momentary assessment questions across hours of day (left) and trial weeks (right).

The hourly and weekly dismissal rates are shown in Figure 3. When looking at time of day, 11 AM, 4 PM, and 7 PM had the highest dismissal rates (Figure 3). These periods also had a number of questions presented (Figure 2). Questions asked at 9 PM and 10 PM had the lowest dismissal rate; users were more likely to answer questions that were presented in the evening (Figure 3). For example, the dismissal rate at 9 PM was significantly lower than the dismissal rate at 11 AM (χ^2^_1_=21.4, *P*<.001); questions asked at 9 PM (10% dismissal rate) were 5 times more likely to be answered than questions asked at 11 AM (50% dismissal rate). The dismissal rate for questions was high at the start and end of the trial and was lowest in the middle of the trial (Figure 3). For example, the dismissal rate in trial week 8 was significantly lower than that in week 2 (week 8: 14.1%; week 2: 54.8%; χ^2^_1_=19.2, *P*<.001).

**Figure 4:**
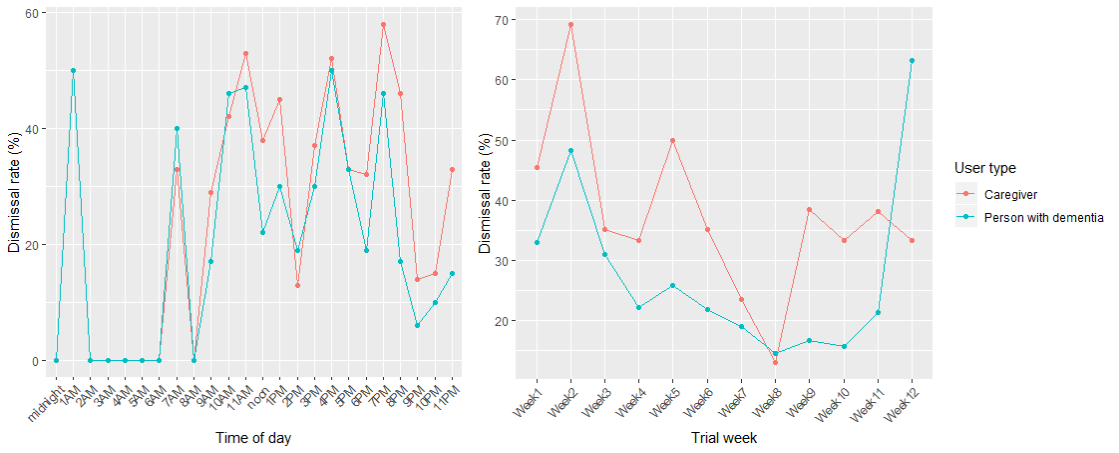
Dismissal rate for ecological momentary assessment questions across hours of day (left) and trial weeks (right) for each user type.

Dismissal rates by time of day and by week for each user type are shown in Figure 4. When looking at time of day, user types demonstrated a similar pattern of dismissals (Figure 4). There was a strong correlation between dismissal rate and time of day between persons with dementia and their caregivers (*P*<.001, ρ=0.81). Weekly dismissal rates differed between user types (Figure 4). There was no correlation between dismissal rate and trial week between persons with dementia and their caregivers (*P*=.09, ρ=0.51).

There was a strong correlation between the number of questions presented and the number of questions dismissed per hour for caregivers (*P*<.001, ρ=0.84) and persons with dementia (*P*<.001, ρ=0.83). From investigating the residuals, there were significantly less questions dismissed at the hours of 2 PM , 9 PM, and 10 PM for both caregivers and persons with dementia. For both user types, questions in the evening were more likely to be answered than dismissed.

### Engagement Following Reminiscence Media Types

The number presented and dismissal rates of questions following reminiscence within photo, video, and music are shown in Table 3. The highest number of questions were presented after users viewed photos, and the fewest after listening to music. Personal media was used in the app more than generic media was used, thus more questions were presented following reminiscence with personal media.

As shown in Table 3, chi-square tests comparing generic to personal media source indicated significant differences when comparing the proportion of questions of dismissed with the proportion of questions answered for generic photos versus personal photos (χ^2^_1_=19.4, *P*<.001) and generic music versus personal music (χ^2^_1_=15.3, *P*<.001). There was no significant difference between proportion of questions of dismissed and answered between generic videos and personal videos (χ^2^_1_=0.2, *P*=.67).

Significantly more questions were dismissed after viewing any type of photo than after listening to any type of music (all photo: 34.4%; all music: 19.3%; χ^2^_1_=10.9, *P*<.001). The dismissal rate for questions asked after viewing personal photos (41/122, 33.6%) was significantly higher than the rates of those asked after generic music (χ^2^_1_=73.7, *P*<.001), personal music (χ^2^_1_=19.2, *P*<.001), and generic photos (χ^2^_1_=19.4, *P*<.001). The dismissal rate for questions asked after interacting with generic music (4/73, 5.5%) was significantly lower than those of personal music (χ^2^_1_=15.3, *P*<.001), personal videos (χ^2^_1_=12.1, *P=*.02), or personal photos (χ^2^_1_=73.7, *P*<.001). These results show that questions asked following reminiscence with personal media, especially those asked after personal photos, were less likely to be answered compared to those asked after other media. In contrast, questions asked after the user had listened to generic media, in particular those asked after generic music, were much more likely to be answered.

**Table 3:** Question presentation and dismissal following reminiscence with different media.

Media type (format and source, questions presented)		Questions dismissed, n (%)	Comparison between generic and personal	
			Chi-square (*df*)	*P* value
**Photo**			19.4 (1)	<.001
	Generic (n=14)	2 (15.4)		
	Personal (n=122)	41 (33.6)		
	All (n=256)	88 (34.4)		
**Video**			0.2 (1)	.67
	Generic (n=2)	0 (0.0)		
	Personal (n=11)	5 (45.5)		
	All (n=57)	16 (28.1)		
**Music**			15.3 (1)	<.001
	Generic (n=73)	4 (5.5)		
	Personal (n=39)	39 (35.9)		
	All (n=176)	34 (19.3)		

### Engagement Following Other App Activities

Questions were also presented to users as they were completing other logged activities. There were significantly more questions answered than were dismissed when a person with dementia logged into the app (answered: 15/20, 75.0%; dismissed: 5/20, 25.0%; χ^2^_1_=8.1, *P*=.004). There was no significant difference between questions answered and questions dismissed when caregivers logged in (answered: 9/14, 64.3%; dismissed: 5/14, 35.7%; χ^2^_1_=1.3, *P*=.26), when caregivers added or deleted photos (answered: 21/43, 48.8%; dismissed: 22/43, 51.2%; χ^2^_1_=0, *P*>.999), or when caregivers or persons with dementia exited the app (answered: 35/61, 57.4%; dismissed: 26/61, 42.6%; χ^2^_1_=2.1, *P*=.15).

## Discussion

### Principal Findings

This study set out to explore engagement with EMA using a case study of a reminiscence app for persons with dementia and their caregivers. Overall engagement with EMA was high, with 69.1% questions answered when they were presented. Other studies [18] have reported between 55% and 87% engagement with EMA questions in the general population. Previous studies [19,20] that have used EMA with older adults have generally relied on sending user notifications or alarms as reminders to complete questions. In these cases, the most commonly reported reasons for not answering EMA questions included being prompted at inconvenient times or when busy, notification sound was not heard, or phone was not nearby at the time [19,20]. In our study, questions were asked only when the users where already using the app which eliminated some of the previously reported reasons for EMA nonadherence.

When trying to increase engagement with EMA within a digital intervention, one important factor to consider is the frequency of administering questions. For example, if EMA questions are presented multiple times in one day it may become burdensome for the user and increase the chances of dismissal. In our study, persons with dementia and their caregivers were less likely to dismiss EMA questions asked between 9 PM and 10 PM. This is similar to what we found in a previous study [21] exploring the temporal behavior of users completing EMA in a maternal health app, where users were more likely to complete mental health scales around 8 PM or 9 PM. Additionally, dismissal rates around 9 AM, 2 PM, and 6 PM which corresponded to postbreakfast, postlunch, and postevening mealtimes were also low suggesting users were more likely to engage in questions during postprandial reminiscence.

Dismissal rates for EMA after reminiscence with generic media were very low when compared to personal media. It has been shown that reminiscence with personal media is more likely to have positive psychosocial benefits compared to generic reminiscence material for persons with dementia [22]. This would suggest that personal media provides a more meaningful reminiscence experience, and thus, could explain why users were less likely to answer questions during this time; however, our results suggest that asking questions following reminiscence with personal photos compared to other media sources was likely to result in question dismissal. We also found that users were less likely to dismiss EMA questions following reminiscence with generic music compared to video or photos. Cognitive function has been shown to be better in persons with dementia following music therapy [23,24]. Therefore, we suggest that participants were more likely to answer questions after listening to music as this was less cognitively demanding compared to viewing photos or video. Music therapy has been shown to have other benefits for persons with dementia, improving behavioral and psychological symptoms and decreasing agitation [25-27]. This could also explain why participants were more likely to answer questions after listening to music.

When caregivers were adding or deleting photos, the dismissal rate was high. Questions asked while users were trying to complete a task such as this could be a hindrance, therefore lead to a higher dismissal rate. Overall, both users were more likely to answer questions if they were asked less frequently so this should be taken into consideration going forward.

Future work should look at the type of EMA questions being asked, for example it may be more beneficial to ask questions such as “Do you find reminiscence therapy helpful?” or “What is your favorite way to reminisce?” rather than questions with Likert scale responses. Alternatively, EMA could assess feedback on the user experience as this would provide co-design opportunities in the use phase since most cocreation activities focus on initial design. These questions could help inform app design and prrovide valuable insight into user experience.

Ultimately, to increase engagement with EMA, it is important to secure individuals as regular app users. There are several approaches which can be used to secure people as full adopters of an app, such as user notifications and prompts to encourage app usage. These notifications could be sent when individuals are likely to engage with EMA, such as in the evening at 9 PM. Personalization can also help to increase engagement. In our study, EMA questions from the mutuality scale incorporated the name of caregiver or person living with dementia, for example, “How attached are you to [partners name]?”. Future work should continue to utilize these personalized aspects such as using individual names in personalized push notifications or in EMA questions.

### Limitations

The main limitation of our study was lack of generalizability to a larger population given the quasi-experimental design and older demographic where half of the participants were persons with dementia. Due to the relatively small sample size, it was not possible to attribute any findings to gender differences. Ideally, sample size should be calculated for a randomized controlled trial to ensure adequate power; however, the sample size for this study (n=56) was deemed sufficient to meet the objectives of a feasibility study.

Another limitation was the recording of log data. User events were logged in real time and were later collected in person from the iPads using portable storage devices. As a result, we could not control for lost local data due to operating system failures, app crashes, bugs, and updates. This was the case with some of the log data which was recorded. For 16% of all EMA questions asked, the event which was logged previously was also an EMA question instead of one of the actions (Multimedia Appendix 2) that should trigger a question being asked. To allow for remote event logging and to minimize data loss, it is important to follow best practices for the collection and storage of user log data, such as storing data in the cloud. This would enable the analysis of logs over the duration of the study. Remote logging could also facilitate the use of adaptive features to motivate persons with dementia and caregivers by sending personalized notifications and motivational messages when app usage is low.

### Conclusions

This study explored engagement with EMA questions using an app for reminiscence where the users were caregivers and persons with dementia. Our results show that older people are willing to participate and engage in EMA; however, more can be done to increase engagement. Notwithstanding the limitations, based on this study we propose a set of recommendations for the use of EMA to optimize user engagement within a digital intervention. EMA questions should add value and help to validate the use of the digital health app in line with the study objectives. It is important that EMA questions do not distract or interfere with the overall purpose of the app which was to allow persons with dementia and their caregivers to reminisce. To avoid overprompting the user, no more than one question should be presented per day and ideally in the evening after 8 PM as this is when people were most likely to engage. If the user is trying to complete a task within the app such as adding content then a question should not be presented. These recommendations can be broadly applied to EMA use in similar settings. Future work will be carried out to study the engagement on a larger scale with more participants, which will further support these recommendations. Future work will also involve studying app usage patterns, for example, if a user engages in an EMA question, when will they next engage, and can we predict engagement based on user log analysis.

## Appendix

Multimedia Appendix 1 Screenshots of the InspireD app.

Multimedia Appendix 2 List of actions completed by app users which could prompt an EMA question.

## Abbreviations

EMA: ecological momentary assessment
InspireD: individual specific reminiscence in dementia

## References

[1] (2019). Dementia. World Health Organisation.

[2] Prince M, Knapp M, Guerchet M, McCrone P, Prina M, Comas-Herrera A (2014). Dementia UK: Second edition – Overview. UK Alzheimer Soc London.

[3] Lewis F, Schaffer SK, Sussex J, O'Neill P, Cockcroft L (2014). The Trajectory of Dementia in the UK - Making a Difference. Office of Health Economics.

[4] Gonzalez J, Mayordomo T, Torres M, Sales A, Meléndez JC (2015). Reminiscence and dementia: a therapeutic intervention. Int Psychogeriatr.

[5] Spector A, Orrell M, Davies S, Woods R T (2000). Reminiscence therapy for dementia. Cochrane Database Syst Rev.

[6] Gibson F (2004). The Past in the Present: Using Reminiscence in Health and Social Care.

[7] Yu F, Mathiason MA, Johnson K, Gaugler JE, Klassen D (2019). Memory matters in dementia: efficacy of a mobile reminiscing therapy app. Alzheimers Dement (N Y).

[8] Woods B, O'Philbin L, Farrell EM, Spector AE, Orrell M (2018). Reminiscence therapy for dementia. Cochrane Database Syst Rev.

[9] Shiffman S, Stone AA, Hufford MR (2008). Ecological momentary assessment. Annu Rev Clin Psychol.

[10] Lazzari C (2018). Ecological momentary assessments and interventions in Alzheimer's caregiving. Curr Alzheimer Res.

[11] Pihet S, Moses Passini C, Eicher M (2017). Good and bad days: fluctuations in the burden of informal dementia caregivers, an experience sampling study. Nurs Res.

[12] Archbold PG, Stewart BJ, Greenlick MR, Harvath T (1990). Mutuality and preparedness as predictors of caregiver role strain. Res Nurs Health.

[13] Bech P, Olsen LR, Kjoller M, Rasmussen NK (2003). Measuring well-being rather than the absence of distress symptoms: a comparison of the SF-36 Mental Health subscale and the WHO-Five Well-Being Scale. Int J Methods Psychiatr Res.

[14] Spruytte N, Van AC, Lammertyn F, Storms G (2002). The quality of the caregiving relationship in informal care for older adults with dementia and chronic psychiatric patients. Psychol Psychother.

[15] Mulvenna M, Gibson A, McCauley C, Ryan A, Bond R, Laird L, Curran K, Bunting B, Ferry F (2017). Behavioural usage analysis of a reminiscing app for people living with dementia and their carers. https://dl.acm.org/doi/10.1145/3121283.3121289.

[16] Laird EA, Ryan A, McCauley C, Bond RB, Mulvenna MD, Curran KJ, Bunting B, Ferry F, Gibson A (2018). Using mobile technology to provide personalized reminiscence for people living with dementia and their carers: appraisal of outcomes from a quasi-experimental study. JMIR Ment Health.

[17] McCauley CO, Bond RB, Ryan A, Mulvenna MD, Laird L, Gibson A, Bunting B, Ferry F, Curran K (2019). Evaluating user engagement with a reminiscence app using cross-comparative analysis of user event logs and qualitative data. Cyberpsychol Behav Soc Netw.

[18] Cain AE, Depp CA, Jeste DV (2009). Ecological momentary assessment in aging research: a critical review. J Psychiatr Res.

[19] Ramsey AT, Wetherell JL, Depp C, Dixon D, Lenze E (2016). Feasibility and acceptability of smartphone assessment in older adults with cognitive and emotional difficulties. J Technol Hum Serv.

[20] Liu H, Lou VWQ (2019). Developing a smartphone-based ecological momentary assessment protocol to collect biopsychosocial data with community-dwelling late-middle-aged and older adults. Transl Behav Med.

[21] Bond R, Moorhead A, Mulvenna M, O'Neill S, Potts C, Murphy N (2019). Exploring temporal behaviour of app users completing ecological momentary assessments using mental health scales and mood logs. Behav Inf Technol.

[22] Subramaniam P, Woods B (2012). The impact of individual reminiscence therapy for people with dementia: systematic review. Expert Rev Neurother.

[23] Bruer RA, Spitznagel E, Cloninger CR (2007). The temporal limits of cognitive change from music therapy in elderly persons with dementia or dementia-like cognitive impairment: a randomized controlled trial. J Music Ther.

[24] Gómez Gallego M, Gómez García J (2017). Music therapy and Alzheimer's disease: Cognitive, psychological, and behavioural effects. Neurologia.

[25] Raglio A, Bellelli G, Traficante D, Gianotti M, Ubezio MC, Villani D, Trabucchi M (2008). Efficacy of music therapy in the treatment of behavioral and psychiatric symptoms of dementia. Alzheimer Dis Assoc Disord.

[26] Sung H, Chang AM (2005). Use of preferred music to decrease agitated behaviours in older people with dementia: a review of the literature. J Clin Nurs.

[27] McDermott O, Orrell M, Ridder HM (2014). The importance of music for people with dementia: the perspectives of people with dementia, family carers, staff and music therapists. Aging Ment Health.

