# Supplementary figures and images for: Ecological Momentary Assessment Within a Digital Health Intervention for Reminiscence in Persons With Dementia and Caregivers: User Engagement Study
Source: JMIR Mhealth Uhealth. 2020 Jul 6;8(7):e17120. doi: 10.2196/17120 (PMC7381015; doi:10.2196/17120)

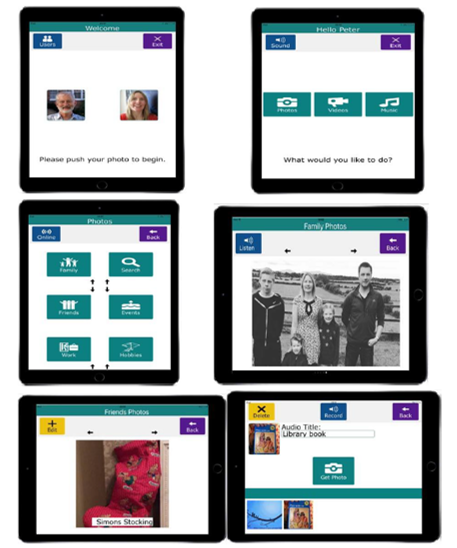

Supplement: Multimedia Appendix 1 [file mhealth_v8i7e17120_app1.png]

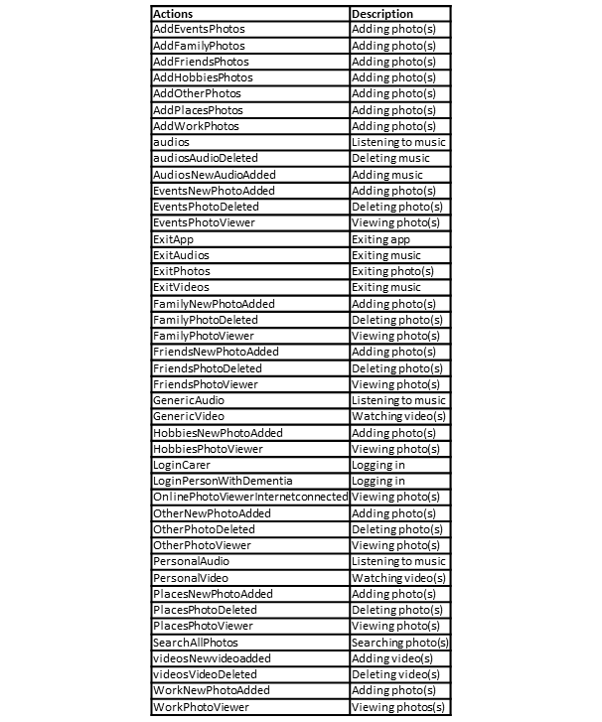

Supplement: Multimedia Appendix 2 [file mhealth_v8i7e17120_app2.png]
